# Supplementary material for: How can digitalization be used to develop community resilience in public health emergencies?: A qualitative comparative analysis from China
Source: PLoS One. 2024 Dec 23;19(12):e0315713. doi: 10.1371/journal.pone.0315713 (PMC11665998; doi:10.1371/journal.pone.0315713)
Supplement: Attachment — Submitted filename: Response to Reviewers.docx [file pone.0315713.s001.docx]

Dear Editorial Department of PLOS ONE:

Hello!

Based on the issues pointed out by the review experts and the proposed modification suggestions, the modification status of this article is briefly described as follows:

Question 1:Please ensure that your manuscript meets PLOS ONE's style requirements, including those for file naming.

Modification 1: Modifications have been made as required.

Question 2:Please state what role the funders took in the study. If the funders had no role, please state: "The funders had no role in study design, data collection and analysis, decision to publish, or preparation of the manuscript."

Modification 2: Explanation of the role of sponsors has been added.

Question3:Please note that your Data Availability Statement is currently missing the repository name and/or the DOI/accession number of each dataset OR a direct link to access each database.

Modification 3: Add a database download link in the cover letter.

Question4：L46: Uncertainty is written twice. Correct it.

Modification4：Correct Uncertainty.

Question5：L133: Remove quotation mark and correct the phrase as uses and used are stated simultaneously (L213 as well).

Revision 5: The quotation marks have been removed and the phrase has been corrected.

Question6：L158, 165: Numbers are overlapping. Try another format.

Revision 6: The issue of overlapping numbers has been resolved.

Question7：L293-294: There are some minor translation errors, digitalization is mentioned twice. Same correction at L430.

Revision 7: Translation errors have been corrected.

Question8：L304: Remove‘initiative was implemented’ since it is already expressed in L303.

Revision 8：already Remove‘initiative was implemented’

Question9：L311,319: Grassroot party building leadership, correct it.

Revision 9: The statement made by grassroots party building leaders has been corrected.

Question10：The authors should consider incorporating terms like "digitalization" or "smart governance" in the title, as this is key to defining the research framework. This would also help readers better understand the study's context.

Revision 10: The term "digitalization" has been added to the title.

Question11：It is recommended that the authors clearly outline the research problem, conclusions, and innovations in the Introduction section.

Revision 11: The research question, conclusion, and innovation have been clearly outlined in the introduction section.

Question12：As the core concept of the paper, the authors should provide a detailed definition of "community resilience." This should include a comparison of community resilience in the context of public health emergencies with its general definition, highlighting both similarities and differences. Additionally, the authors should discuss how their conclusions offer innovative insights compared to existing research on community resilience. This will further emphasize the value of exploring community resilience specifically in the context of public health emergencies.

Revision 12: The community resilience in the context of sudden public health emergencies has been compared with its general definition in the introduction, highlighting the similarities and differences.

Question13：A critical issue to address is how this research might provide insights applicable to other countries and regions beyond China.

Revision 13: The discussion section in the text has already explained how the research can be applied to insights from countries and regions outside of China.

Question14：The choice of five condition variables needs a detailed theoretical or practical justification. Additionally, the authors need to ensure clear differentiation between these variables. For instance, the manuscript uses “the identity of the team leader (provincial party secretary = 4, governor = 3, vice governor = 2, other = 1)” to measure Policy Support. However, in the Chinese context, this variable may also reflect aspects of Leadership and Party-building to some extant. Therefore, the authors should ensure the independence of the variables.

Revise 14: Make indicator adjustments to ensure the independence of variables.

Question15：Clarification is needed on how variables like “Digitization of organizations” are measured. The explanation provided is insufficient: “The degree of digitization in organizations is measured through the digitalization of party and government institutions, as assessed by the “2021 China Digital Government Construction White Paper.” At what level of party and government is it based, and how is this ‘digitalisation’ measured? The authors need to address these questions in more detail. Similarly, the five indicators used to measure “community resilience” should be exemplified to aid reader understanding.

Revision 15: The selection criteria for each indicator have been explained in detail.

Question16：The Discussion section lacks content on the theoretical contributions of the study.

Revision 16: Content regarding the theoretical contribution of this study has been added to the discussion section.

Question17：The Policy Insights section should provide more detailed recommendations on policy or practice. Current suggestions are somewhat broad and lack actionable guidance for practitioners.

Revision 17: More detailed recommendations on policies or practices have been provided in the Policy Implications section.

Question18：The manuscript suffers from numerous language issues. For example: “Community resilience refers to a community's ability of a community to learn from each crisis...”; “The study proposes a 'qualitative' and 'quantitative' approach based on a complex dynamic perspective of histories."”; “The study uses the fsQCA method is used to analyze 31 provinces and cities in mainland China...”; “In this paper, we use data from 31 provincial-level regions in mainland China are selected as the sample.”; “we use the fsQCA method is used to study the factors that...”; “Digital Government The degree of construction of”; “grassroots party-building leadership of grassroots party building.”

Revision 18: Language issues in the manuscript have been corrected.

Question19：There are numerous issues with the translation of references. For instance, "管理世界" should be translated as "Journal of Management World," not “Managing the World,” and "Theoretical Investigation" was incorrectly translated as “LILUN TANTAO.” The authors should carefully correct these errors.

Revision 19: Translation issues with references have been corrected.

Question20：The clarity of the Figures in the manuscript should be improved.

Revision 20: The clarity of numbers in the manuscript has been improved.

Thank you for pointing out the issues and providing valuable revision suggestions. The research team has re proofread and checked the paper according to the editorial department's comments, and the revised parts have been highlighted in red. If there are any further modifications, please let me know. Thank you!

Li Liqing , Li Zihan , Ding Haifeng and Gao Meng

September 14, 2024
