# Supplementary material for: How can digitalization be used to develop community resilience in public health emergencies?: A qualitative comparative analysis from China
Source: PLoS One. 2024 Dec 23;19(12):e0315713. doi: 10.1371/journal.pone.0315713 (PMC11665998; doi:10.1371/journal.pone.0315713)
Supplement: Attachment — Submitted filename: PONE-D-24-10967_review report.docx [file pone.0315713.s002.docx]

**Review Report**

The revised manuscript titled "How can digitalisation be used to develop community resilience in public health emergencies?: A qualitative comparative analysis from China" presents a well-structured and scientifically sound study. The research objectives are clear, the methodology is appropriate, and the results are significant and well-interpreted. The study contributes valuable insights to the field and meets the standards of this journal. However, there are numerous grammatical mistakes, translation and typo errors throughout the manuscript, making it difficult to recommend it in its current form for publication.

1. There are still numerous grammatical and typo errors. Line 108 typo error (“), Line 139 (,), and similar issues can be seen throughout the manuscript. Please review thoroughly and correct such errors.
2. Figure 1 is missing. It does not show in this manuscript.
3. I suggest it is best to revise all phrases related to data sample areas as 31 provincial-level regions or 31 provinces or 31 cities to avoid confusions.
4. Line 161 states configuration theory as theoretical framework for "holistic intelligent governance - community resilience formation", while Line 182-184 states holistic governance theory and digital governance theory as theoretical framework for “holistic wisdom governance-community resilience formation”. Clarify the theoretical framework and use one term “wisdom/intelligent” throughout the manuscript for better readability.
5. There are typo errors in references sections. Add space between words, check reference 4,8,9,10…

I find the manuscript requires minor revision.
