# Supplementary material for: How can digitalization be used to develop community resilience in public health emergencies?: A qualitative comparative analysis from China
Source: PLoS One. 2024 Dec 23;19(12):e0315713. doi: 10.1371/journal.pone.0315713 (PMC11665998; doi:10.1371/journal.pone.0315713)
Supplement: Attachment — Submitted filename: Response to Reviewers.docx [file pone.0315713.s003.docx]

**Response letter**

**Dear Editor,**

We would like to thank you and all reviewers for your insightful comments concerning the revisions of our manuscript entitled “***How can digitalization be used to develop community resilience in public health emergencies?: A qualitative comparative analysis from Chinas***”. We have carefully reviewed the reviewers’ comments, and we have addressed all their questions/critiques/concerns point-by-point as below. Mark all changes in tracked so that they may be easily identified. We believe that our manuscript has been significantly improved, and we hope it is now suitable for publication.

If you have any problems with our manuscript, please do not hesitate to contact us.

**Review Report**

The revised manuscript titled "How can digitalisation be used to develop community resilience in public health emergencies?: A qualitative comparative analysis from China" presents a well-structured and scientifically sound study. The research objectives are clear, the methodology is appropriate, and the results are significant and well-interpreted. The study contributes valuable insights to the field and meets the standards of this journal. However, there are numerous grammatical mistakes, translation and typo errors throughout the manuscript, making it difficult to recommend it in its current form for publication.

1. There are still numerous grammatical and typo errors. Line 108 typo error (“), Line 139 (,), and similar issues can be seen throughout the manuscript. Please review thoroughly and correct such errors.

Response: Thank you for your feedback. We have carefully reviewed the manuscript and corrected all grammatical, typographical, and punctuation errors, including those in Line 108 and Line 139.

2. Figure 1 is missing. It does not show in this manuscript.

Response: Thank you for pointing this out. Figure 1 has been uploaded separately in the system according to the journal's submission requirements and was not included in the main text.

3. I suggest it is best to revise all phrases related to data sample areas as 31 provincial-level regions or 31 provinces or 31 cities to avoid confusions.

Response: Thank you for your suggestion. We have revised the manuscript to consistently use the term "31 provincial-level regions in mainland China" to avoid confusion.

4. Line 161 states configuration theory as theoretical framework for "holistic intelligent governance - community resilience formation", while Line 182-184 states holistic governance theory and digital governance theory as theoretical framework for “holistic wisdom governance-community resilience formation”. Clarify the theoretical framework and use one term “wisdom/intelligent” throughout the manuscript for better readability.

Response: Thank you for your valuable comments. We have clarified the theoretical framework and unified the terminology throughout the manuscript. "Holistic intelligent governance" is now consistently used instead of varying terms.

5. There are typo errors in references sections. Add space between words, check reference 4,8,9,10…

Response: Thank you for pointing this out. We have thoroughly reviewed the references section and corrected the typos, ensuring proper spacing between words and fixing references 4, 8, 9, and 10.

**Reviewer #2:** I appreciate the authors’ effective improvements and their appropriate responses to some of my concerns in this round of revisions, which is commendable. However, to enhance the rigor of the manuscript and increase its chances of acceptance, the authors should address the following points:

1. Given that PLOS ONE is an international journal aimed at a global audience, I recommend that the authors remove certain culturally specific background elements related to China, such as references to specific government meetings and speeches by national leaders, as well as particular phrases like “modernizing the national governance system and capacity.”

Response: Thank you very for your suggestions. This study mainly employs the fuzzy-set qualitative comparative analysis (fsQCA) method to establish a configuration research framework for the promotion of community resilience through holistic intelligent governance in response to public health emergencies. Using 31 provincial-level regions in mainland China as the research sample, we explore the configuration patterns and development paths of holistic intelligent governance that foster community resilience, all within the context of Chinese scenarios and issues. We sincerely appreciate your advice, and in our future research, we will broaden the scope of our study to include a global perspective.

2. Please review the clarity of the text, such as the phrasing in line 139: “of grassroots governance [19].,”

Response: Thank you for your feedback. The phrasing in line 139 has been revised for clarity.

3.I suggest relocating the literature review from the “Introduction” section to a later chapter to address the redundancy in the first section.

Response: Thank you for your suggestion. The introduction section was indeed lengthy, but it contains a description of the research background, which helps readers understand the context, continuity, and relevance of the study. It also ensures that the scientific rigor and feasibility of the research are clear to the readers. Additionally, the journal's submission guidelines require the introduction to be placed at the beginning. We appreciate your input, and in order to avoid excessive length, we have made some reductions to the introduction. Once again, thank you for your valuable feedback.

4.The authors should also carefully verify the accuracy of the translations of cited Chinese literature, as I pointed out in the first round of feedback. For instance, the correct translation of “清华管理评论” is “Tsinghua Business Review,” not “Tsinghua Management Review.” I encourage the authors to ensure that each reference is accurately translated.

Response: Thank you for your valuable feedback. We have carefully reviewed the translations of the cited Chinese literature and have made the necessary corrections, including updating “清华管理评论” to "Tsinghua Business Review." We will ensure that all references are accurately translated.
